# Stability of Adult Emergence and Activity/Rest Rhythms in Fruit Flies *Drosophila melanogaster* under Semi-Natural Condition

**DOI:** 10.1371/journal.pone.0050379

**Published:** 2012-11-28

**Authors:** Nisha N. Kannan, Vishwanath Varma, Joydeep De, Vijay Kumar Sharma

**Affiliations:** Chronobiology Laboratory, Evolutionary and Organismal Biology Unit, Jawaharlal Nehru Centre for Advanced Scientific Research, Jakkur, Bangalore, Karnataka, India; Washington University Medical School, United States of America

## Abstract

Here we report the results of a study aimed at examining stability of adult emergence and activity/rest rhythms under semi-natural conditions (henceforth SN), in four large outbred fruit fly *Drosophila melanogaster* populations, selected for emergence in a narrow window of time under laboratory (henceforth LAB) light/dark (LD) cycles. When assessed under LAB, selected flies display enhanced stability in terms of higher amplitude, synchrony and accuracy in emergence and activity rhythms compared to controls. The present study was conducted to assess whether such differences in stability between selected and control populations, persist under SN where several gradually changing time-cues are present in their strongest form. The study revealed that under SN, emergence waveform of selected flies was modified, with even more enhanced peak and narrower gate-width compared to those observed in the LAB and compared to control populations in SN. Furthermore, flies from selected populations continued to exhibit enhanced synchrony and accuracy in their emergence and activity rhythms under SN compared to controls. Further analysis of zeitgeber effects revealed that enhanced stability in the rhythmicity of selected flies under SN was primarily due to increased sensitivity to light because emergence and activity rhythms of selected flies were as stable as controls under temperature cycles. These results thus suggest that stability of circadian rhythms in fruit flies *D. melanogaster*, which evolved as a consequence of selection for emergence in a narrow window of time under weak zeitgeber condition of LAB, persists robustly in the face of day-to-day variations in cycling environmental factors of nature.

## Introduction

Circadian rhythms are manifestation of an organism’s temporal adaptation to daily changes in cyclic environmental conditions. Therefore, the accuracy with which circadian timing systems maintain stable phase-relationship with cyclic environment is likely to be a key determinant of the organism’s temporal adaptation in nature [1]. While it is likely that a large variety of time-cues (zeitgebers) have contributed to the evolution of circadian rhythms, most organisms seem to use light/dark (LD) cycles as the primary zeitgeber to time their cyclic behavioural and metabolic processes [2]. Besides LD cycles, circadian clocks of some organisms have been shown to synchronize to warm/cold (WC) cycles in otherwise constant laboratory condition [3]–[5]. While studies to examine circadian entrainment have invariably been carried out in the laboratory under simulated natural LD and WC cycles, such conditions markedly differ from natural environment in several aspects, including abrupt versus gradual changes in environmental factors, stochasticity and levels of these factors [6]–[13]. A few recent studies in fruit flies *Drosophila melanogaster* attempted to elucidate circadian entrainment of adult emergence and activity/rest rhythms under natural conditions [13], [14]. During summers, flies show a third peak of activity, occurring during hottest and brightest part of the day, manifested in an additional “afternoon peak” [13]. Adult emergence under SN occurs mostly during dawn coinciding closely with humidity maxima and temperature minima of the day [14]. The gate-width of emergence, variance in peak-to-peak timing and fraction of flies emerging during night were shown to be modulated considerably in different seasons. The major differences in emergence rhythm between semi-natural (SN) and laboratory (LAB) conditions include much narrower emergence gate-width and reduced nighttime emergence in SN along with the finding that rhythmicity in emergence is rescued in genetically arrhythmic *period* null (*per^0^*) flies [14]. Therefore, from these studies it is clear that several aspects of daily rhythms differ between natural and laboratory conditions. This raises concern about the validity of deductions made about rhythmic behaviours in nature based on laboratory studies, and whether they can be used to draw inferences regarding the evolutionary forces behind the origin of circadian clocks. For instance, a recent study asked whether early and late emergence chronotypes which evolved as a result of selection for morning and evening emergence under weak environmental cycles of the laboratory would persist in nature [15]. Surprisingly, not only early-late chronotypes persisted in these populations, under semi-natural conditions the phenotypes became even more striking, and showed no correlation with natural morning and evening transitions, unlike that observed under laboratory conditions. Such differential effect of LAB versus SN on the phase of emergence makes it relevant to ask how stability of circadian rhythms is influenced by the presence of multiple gradually changing zeitgebers of nature.

It is expected that precisely timed rhythmic phenomena would confer greater adaptive advantage to organisms living in cyclic environment compared to randomly occurring ones [16], [17]. Furthermore, findings from some studies show association between circadian period close to 24 h and higher precision, suggesting that stability in circadian entrainment contributes to the evolution of circadian clocks in the face of cyclic fluctuations in nature [18]–[20]. To critically examine this, we carried out a laboratory selection study, where we imposed selection for adult emergence in a narrow window of time. As a result, selected populations evolved enhanced synchrony and accuracy in the phase of entrainment under LD cycles, and increased homogeneity and precision in clock period under constant darkness (DD) [21]. Furthermore, flies from selected populations displayed enhanced stability in circadian entrainment under a wide variety of environmental conditions created in the laboratory [22]. Although all our assessments of stability in circadian entrainment were performed under laboratory condition aided with relatively weak entraining cues - LD cycles with a light intensity of ∼100 lux and constant temperature, humidity and *ad libitum* food, these studies clearly indicate that stability in circadian time-keeping evolves in response to periodic selection pressures under cyclic environment. We examined whether the mechanisms that evolve under the influence of weak selection pressures in the laboratory, contribute in any way to render the selected populations in having stable rhythmic behaviours under natural conditions which are intrinsically stronger, yet more labile. Moreover, the instance of reduction or enhancement of the differences in stability between selected and control populations under SN, could provide clues for the distinctions and relative contributions of environmental factors in determining stable circadian entrainment.

Stability of rhythmic behaviours in nature is thought to be determined by the interplay of stability of circadian pacemakers and of entraining signals [19]. Therefore, assessment of stability in circadian entrainment under SN is likely to provide a more realistic picture of how stability of rhythmic behaviour is maintained in nature. With this in mind, we examined emergence and activity rhythms of four populations each of selected and control flies under SN and LAB, and used various aspects of rhythms such as amplitude, synchrony and accuracy as measures of stability. To tease apart the role of light and temperature on stability in circadian entrainment, we assayed these rhythms in flies from selected and control populations under laboratory LD and WC cycles. Stability of emergence and activity rhythms of flies from populations selected for precise timing of adult emergence persisted under SN more robustly with higher stability, primarily mediated by light.

## Materials and Methods

### Population Maintenance and Selection Protocol

Four replicate populations were initiated by selecting for flies that emerged during ExT7-8 (External Time 7–8, where time of the day is expressed as time difference in hour relative to the mid-point of dark phase [23]) under 12∶12 h LD cycles. Anticipating evolution of reduced variance in clock properties we had named these populations as Precision Populations (*PP*; [21], [22]). These populations were originated from four ancestral baseline populations of *D. melanogaster* which have been maintained under laboratory for several hundred generations under 12∶12 h LD cycles at 25°C on banana-jaggery (BJ) food [24]. These baseline populations were derived from an ancestral population, namely the Ives population [25], which were wild caught from South Amherst, Massachusetts, USA, about 30 years ago. Those populations were maintained in the laboratory for about 110 generations under constant light (LL), constant temperature (25±1°C) and constant humidity on a 14 day discrete generation cycle. Five replicate populations (B1.B5) were derived from the IV population [26], [27], from which after about 360 generations, 5 sets of UU populations were derived [28]. The UU populations were maintained on a 21 day discrete generation cycle, and reared under LL regime in the laboratory for about 170 generations after which four populations (JB1.JB4) were derived from four UU populations (UU1, 2, 3, 5; [24]) from which after ∼100 generations four LD (1.4) populations were derived. These populations served as the baseline populations for the selection experiments. We positioned the selection window 1 h (ExT7) after lights-on (ExT6) to select flies from emergence peak and to exclude those that emerge in ‘startle response’ to lights coming on. By doing so we ensured that flies that emerge as a result of ‘masking’ of emergence rhythm are not a part of the breeding population for the next generation. Four replicate control populations (*CP_1–4_*) were also initiated along with four replicate selected populations (*PP_1–4_*), in which flies emerging throughout the day were used as breeding population for the next generation. Therefore, *CP* populations experienced all other conditions similar to the selected populations except that they were not under any conscious selection for timing of emergence. For both selected and control populations, flies that emerged for four successive days (9^th^–12^th^ day after egg collection) were taken to form breeding adults for the next generation. A total of 1200 adults per population, with approximately equal number of males and females, were maintained in plexiglass cages of 25×20×15 cm^3^ dimension with BJ medium in petridish as food, which was changed every alternate day. Flies were fed with yeast-acetic-acid paste for 3 days before egg collection to induce greater egg production. Three days later, eggs were collected over 3 h window (ExT7–10) on BJ medium and approximately ∼300 eggs were transferred to glass vials (18 cm height×2.4 cm diameter) containing ∼10 ml of BJ medium. Thus, both selected and control populations were maintained on a 21 day non-overlapping generation cycle. Exactly 48 and 16 such vials were set-up at every generation for each of the *PP* and *CP* stocks, respectively. The selection has being continuing for over 80 generations. Prior to the assays, all selected and control populations were subjected to one generation of common rearing condition to minimize non-genetic parental effects which may have been caused by the imposition of the selection protocol. The progeny of these flies will be henceforth referred to as “standardized flies”.

### SN and LAB Conditions

It is likely that levels of light, temperature and humidity experienced by the flies in glass vials/tubes are different from what was measured by the Drosophila Environmental Monitor (DEnM), and therefore we prefer to refer our outdoor experimental conditions as semi-natural (SN). Condition used in the LAB was either 12∶12 h LD cycles with ∼100 lux light intensity at ∼25°C, or 12∶12 h WC cycles (29/25°C) in constant darkness (DD). The reason behind the choice of light intensity of ∼100 lux for the light phase of LD was because these flies were reared under this light intensity for about 80 generations. Assays under SN were conducted in an outdoor enclosure kept under canopy [14] inside the campus of Jawaharlal Nehru Centre for Advanced Scientific Research, Bangalore (12°59′N 77°35′E). During the assays under SN, light, temperature and humidity were continuously monitored using DEnM System from Trikinetics, USA.

### Adult Emergence Assay

For the adult emergence rhythm assay, eggs were collected from standardized populations and transferred at a density of ∼300 eggs per vial containing ∼10 ml of food. In each environmental regime, 10 such vials were used for each of the four replicate populations, and four such replicates were used for selected and control populations each. Thus, for the adult emergence assay, under each environmental regime, a total of 40 vials were used for *PP* and 40 for *CP* populations. Emergence rhythm of *PP* and *CP* was assayed under LAB and SN conditions during spring (18^th^ to 23^rd^ February 2011). Vials kept under LAB (LD and WC) and SN conditions were closely monitored for the emergence of the first fly and thereafter assayed regularly at every 2 h interval for at least 5 successive days and the number of emerging adults in every 2 h period recorded. To assess the percentage of flies that emerged close to the selection window, we assayed during ExT5-10 the number of adults emerging every 1 h. To estimate the percentage of flies that emerge in 1 or 2 h interval, we calculated the total number of flies that emerged from each vial per cycle for all the five days. To obtain percentage emergence, the number of flies that emerged during 1 or 2 h assay interval was divided by the total number of flies that emerged in that particular cycle. The data from each replicate population were averaged over five consecutive cycles. The mean values obtained from four such replicate populations were plotted as a function of external time [23]. We also estimated the gate-width of emergence by calculating time-interval between the start and end of emergence on each day, with start and end defined as emergence greater than or less than an arbitrary cut-off of 5% of daily emergence.

### Activity/Rest Assay

The activity/rest behaviour of flies was assayed for a minimum of 8–10 days under LAB (LD and WC) and SN conditions during spring (12^th^ to 19^th^ March 2011). For this, eggs were collected from the standardized populations and transferred into vials containing ∼10 ml of food at a density of ∼300 eggs per vial. Two-day old virgin males were introduced singly into activity tubes to record their activity/rest behaviour using Drosophila Activity Monitors from Trikinetics, USA. The activity/rest behaviour of flies was recorded continuously for 8–10 days. Under each environmental regime, about 32 flies were used for each replicate population, and four replicate populations each were used for *PP* and *CP*. The daily waveform of activity rhythm of each fly was obtained by dividing hourly collected activity data by the total amount of activity of that fly in that cycle. The waveform plotted is averaged across four replicate populations each having ∼32 flies. The phase of morning activity peak was taken as the time point in the morning showing maximum activity. The comparisons of afternoon and evening peaks are based on the differences seen in the waveforms.

### Synchrony and Accuracy of Emergence and Activity Rhythms

Synchrony and accuracy was used as measures of inter-population/individual, and intra-population/individual variance in daily rhythm of selected and control populations [21], [29]. To estimate synchrony in emergence rhythm of each replicate population, mean phase-relationship (*ψ*) of the emergence peak relative to lights-on was obtained for each of the 10 vials, by averaging it across 5 cycles. The inverse of standard deviation (SD) across 10 such average *ψ* values was used as a measure of “synchrony” in emergence rhythm of each replicate population. The mean values obtained from four such replicate populations were used to calculate the synchrony of the population. Similarly, mean *ψ* of morning activity peak for each individual fly was obtained by averaging it across 10 cycles. The inverse of SD across 32 such mean *ψ* values was used as a measure of synchrony of activity rhythm within each population. The mean values obtained from four such replicate populations were used to calculate the synchrony of the population. “Accuracy” was estimated as the reciprocal of SD of day-to-day *ψ* of emergence or activity rhythms. To estimate accuracy of emergence rhythm of each replicate population, we first calculated the inverse of SD of daily *ψ* of emergence peak for each vial across 5-days and then averaged it across all 10 vials for each replicate population. The mean values obtained from four such replicate populations were used as “accuracy”. Similarly for activity rhythm, we estimated reciprocal of SD of daily *ψ* of morning activity peak across 10 days for each individual and then averaged it across all flies. Synchrony and accuracy have a unit of h^−1^.

### Statistical Analyses

The emergence and activity during the selection window and their waveforms, gate-width of emergence, synchrony and accuracy of emergence and activity rhythms were analyzed separately in mixed model analysis of variance (ANOVA) treating replicate populations (Block-B) as random factor, environmental condition (E), population (P) and time point (T) as independent fixed factors crossed with replicates. Mean values of emergence at every 1 or 2 h and activity at every 1 h were the dependent variables used as inputs in the mixed model ANOVA. For specific measures, the mean values of each replicate population of the dependent variables such as emergence and activity during the selection window, gate-width of emergence, synchrony and accuracy of emergence and activity rhythms were the inputs for the model. ANOVA was followed up by post-hoc multiple comparisons using Tukey’s test. 95% Comparison Interval (95%CI) calculated using minimum significant difference in Tukey’s test [30] was used in the figures as error bars to facilitate visual hypothesis testing [31]. Therefore, overlapping error bars would imply that the values do not differ statistically. The waveforms of emergence rhythm of *PP* and *CP* flies were additionally analyzed using Kolmogorov-Smirnov two-sample test to assess whether or not they were different. All analyses were implemented on STATISTICA™ for Windows Release 5.0 B [32].

## Results

### Persistence of Robust Emergence Rhythm Under SN

**Figure 1:**
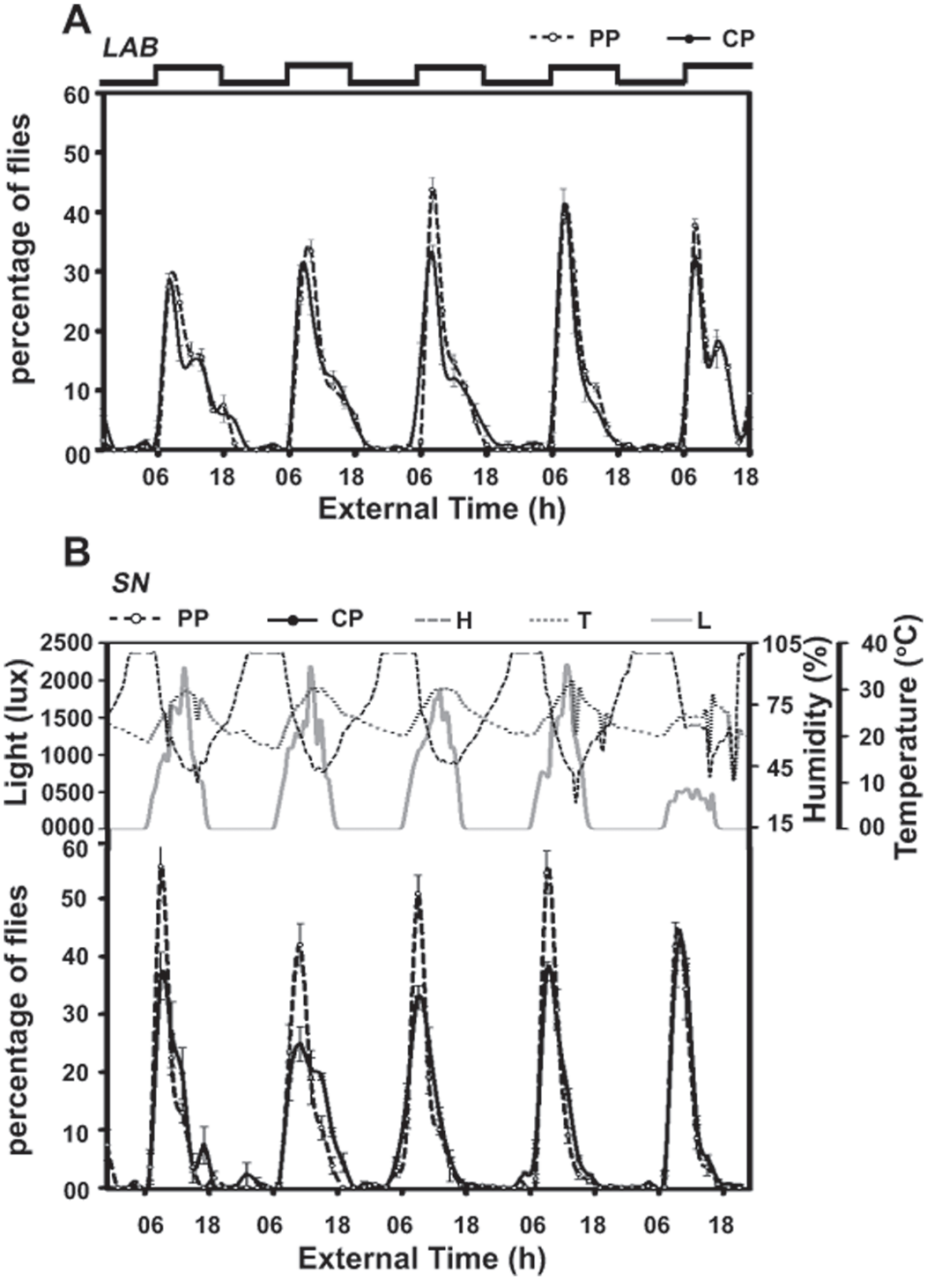
Enhanced robustness in adult emergence of selected population persists under semi-natural (SN). (A) Circadian waveform of emergence of selected (*PP*) and control (*CP*) flies under laboratory (LAB). Percentage of flies that emerged in 2 h bins over five successive cycles is plotted along *y*-axis and External Time (in h) along *x*-axis. The square wave indicated above the figure represents 12∶12 h light/dark (LD) cycles. (B) Circadian waveform of emergence under SN. Percentage of flies that emerged in 2 h bins over five successive cycles are plotted along *y*-axis and External Time in h along *x*-axis. Upper panels of 1B show daily profiles of light (lux), temperature (^°^C) and humidity (%RH) for the entire duration of the experiment. Under LAB, greater percentage of *PP* flies emerged during the peak which persisted under SN. For this assay, ten vials for each of the four replicate populations were used under each environmental condition. The mean values of four replicates and variance in terms of 95% comparison interval (95%CI) were used to draw emergence profiles and error bars.

**Figure 2:**
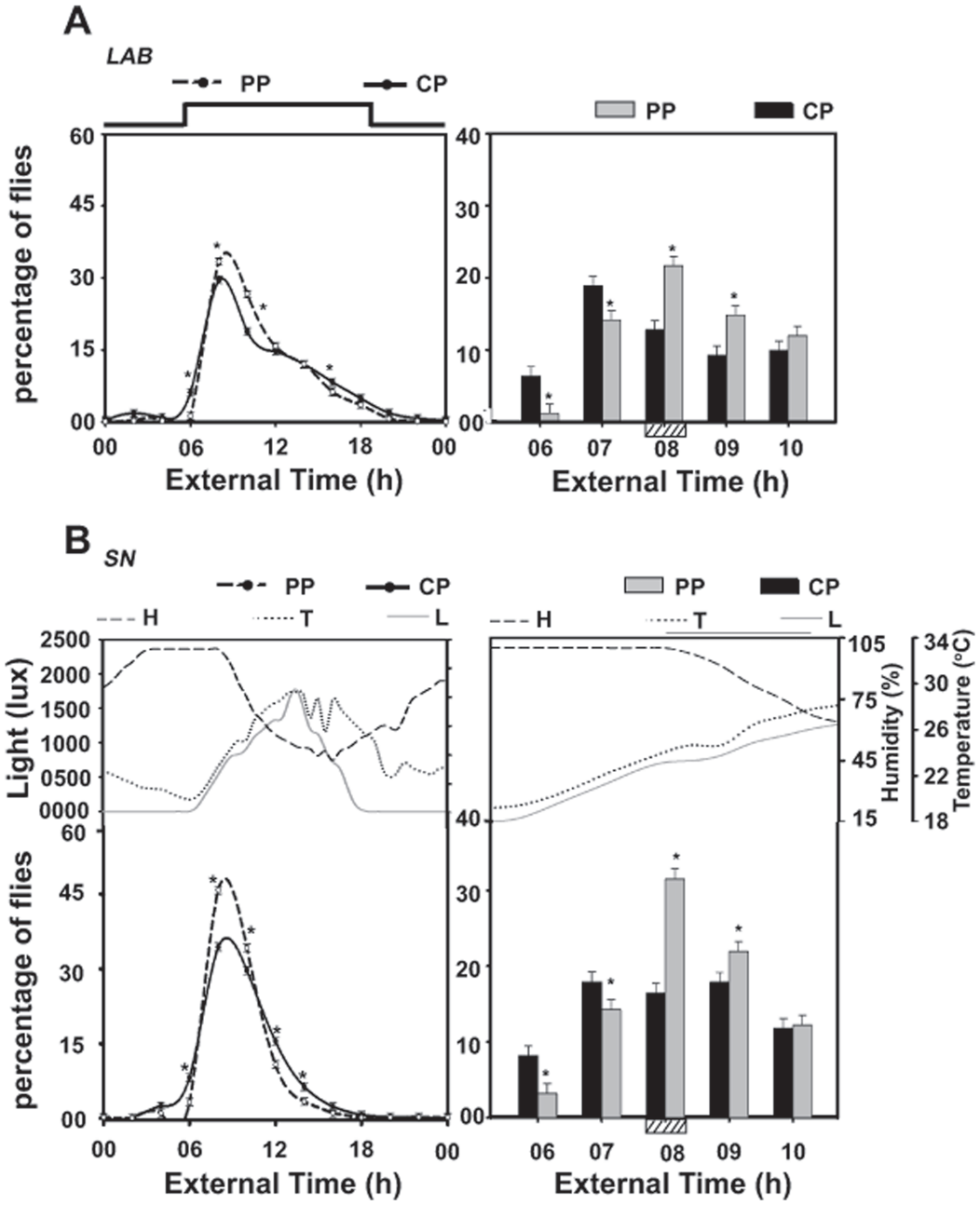
Enhanced stability in adult emergence of selected population persists under semi-natural (SN) condition. (A) Waveform of adult emergence (left panel) in selected (*PP*) and control (*CP*) populations averaged across five consecutive cycles under laboratory (LAB). Percentage of flies emerged in 2 h bins are plotted along *y*-axis and External Time in h along *x*-axis. The square wave line above the Figure represents 12∶12 h light/dark (LD) cycles under laboratory condition. Peak of emergence in *PP* and *CP* populations under LAB (right panel). (B) Waveform of emergence (left panel) of *PP* and *CP* populations averaged across five consecutive cycles under SN. Peak of emergence in *PP* and *CP* populations under SN (right panel). Upper panels of 2B show average profiles of light (lux), temperature (°C) and humidity (%RH) for the entire duration of the experiment. A greater percentage of *PP* flies emerge during the selection window compared to *CP*. Concurrent to the increase in percentage emergence during the selection window there is a decrease in percentage emergence prior-to the selection window, with reduced anticipation to lights-on. Shaded box on the *x*-axis represents the selection window. Under SN, *PP* flies sustain robust waveform of emergence with more prominent peak and narrower gate-width. Error bars represent 95% comparison intervals (95%CI) around the mean for visual hypothesis testing. For this assay, ten vials for each of the four replicate populations were used under each environmental condition. The mean values of four replicates and variance in terms of 95% comparison interval (95%CI) were used to draw emergence profiles and error bars.

**Figure 3:**
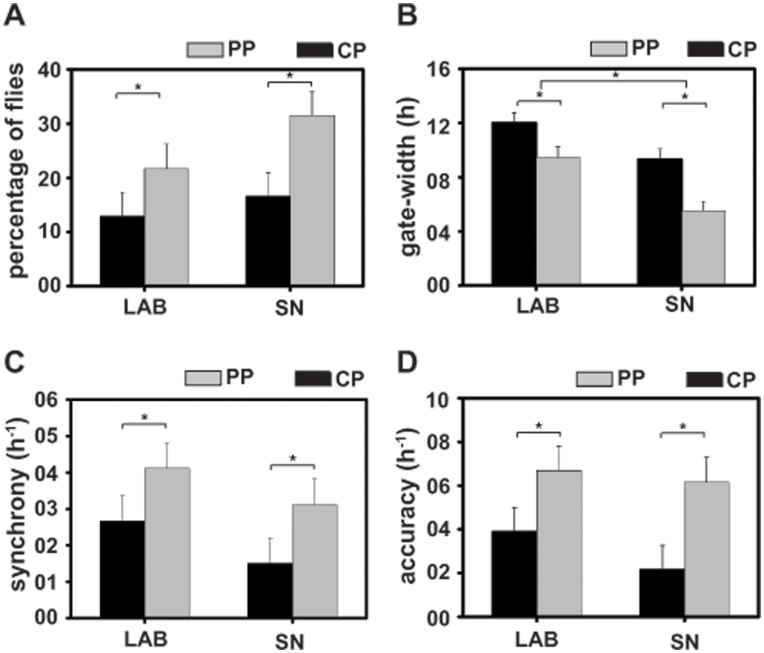
Selected flies in semi-natural (SN) condition emerge in a narrower gate with enhanced accuracy. (A) Percentage of flies emerged during the selection window (ExT7-8) under laboratory (LAB) and SN. Higher percentage of flies emerges during the selection window under LAB as well as SN. (B) Gate-width of emergence rhythm in selected (*PP*) and control (*CP*) populations. *PP* flies exhibited narrower emergence gate under SN. (C, D) Synchrony and accuracy of *PP* and *CP* flies under LAB and SN. *PP* flies exhibit greater synchrony and accuracy compared to controls under both conditions. All other details are same as in Figure 2.

As we have shown previously [21], under LAB, *PP* showed enhanced peak of emergence compared to *CP*, and this difference persisted even more robustly under SN (Figures 1, 2B). In addition, daily emergence waveform of *PP* under SN was more robust than *CP*, marked by enhanced emergence peak and reduced gate-width (Figures 1B, 2B, 3B). This was further confirmed in Kolmogorov-Smirnov’s test, which revealed that while emergence waveform of *PP* and *CP* was significantly different from each other under both LAB (*p*<0.05) as well as SN conditions (*p*<0.01), emergence waveform of each both *PP* (*p*<0.05) and of *CP* (*p*<0.05) were significantly different when compared among LAB and SN.

While more *PP* flies emerged during the selection window (ExT7-8) compared to *CP* under both conditions (Figures 2A, B, 3A), *PP* flies emerged in greater numbers during the selection window under SN than in LAB. Concurrent to increase in percentage of *PP* flies emerging during selection window, a decrease prior-to (ExT6-7) and an increase after (ExT8-9), associated with reduced lights-on anticipatory emergence was observed under both conditions (ExT5-6; Figure 2A, B). ANOVA on emergence data revealed a statistically significant effect of T (*F_11,33_* = 416.4; *p*<0.0001), E×T (*F_11,33_* = 39.3; *p*<0.0001), P×T (*F_11,33_* = 185.8; *p*<0.0001), E×P×T interaction (*F_11,33_* = 152.3; *p*<0.0001), while the effect of E (*F_1,3_* = 0.8; *p* = 0.4) and P was statistically not significant (*F_1,3_* = 0.8; *p* = 0.4). Post-hoc multiple comparisons using Tukey’s test revealed that while under both conditions, percentage of *PP* flies emerging during selection window was significantly higher compared to *CP*, under SN, *PP* emerged in greater numbers compared to LAB (Figure 3A). Enhanced amplitude of emergence rhythm in *PP*, suggests that under SN emergence rhythm of *PP* was more robust than *CP*.

### Selected Flies have a Narrower Gate of Emergence Under SN

Under LAB, selected populations had a significantly reduced gate-width compared to controls, which persisted even more robustly under SN (Figures 2A, 3B), with gate-width difference between *PP* and *CP* was greater compared to that seen in LAB (Figures 2A, B, 3B). ANOVA on gate-width data revealed a statistically significant effect of E (*F_1,3_* = 179.6; *p*<0.0008), P (*F_1,3_* = 153.9; *p*<0.001) and E×P interaction (*F_1,3_* = 5.64; *p*<0.04). Post-hoc multiple comparisons using Tukey’s test revealed that gate-width of emergence in both populations was reduced significantly under SN compared to LAB, which suggests that emergence rhythm was more robust under SN, however, under both conditions gate-width of *PP* flies was significantly narrower compared to *CP* (Figure 3B).

### Selected Flies Display Enhanced Synchrony and Accuracy in Emergence Rhythm Under SN

The synchrony and accuracy of emergence rhythm of *PP* was significantly higher compared to *CP* (Figure 3C, D). ANOVA revealed a statistically significant effect of E (synchrony-*F_1,3_* = 15.81; *p*<0.02; accuracy-*F_1,3_* = 17.54; *p*<0.02) and P (synchrony-*F_1,3_* = 19.2; *p*<0.02; accuracy-*F_1,3_* = 38.2; *p*<0.008), while the effect of E×P interaction was statistically not significant (synchrony-*F_1,3_* = 1.1; *p* = 0.3; accuracy-*F_1,3_* = 0.73; *p* = 0.4). Post-hoc multiple comparisons using Tukey’s test revealed that, under both conditions synchrony and accuracy of emergence rhythm were significantly higher in *PP* compared to *CP* (Figure 3C, D), but of a given population did not differ between SN and LAB. Persistence of enhanced synchrony and accuracy indicates that emergence rhythm of selected flies continues to be more stable under SN compared to controls.

### Selected Flies Display Enhanced Morning Peak of Activity Under SN

**Figure 4:**
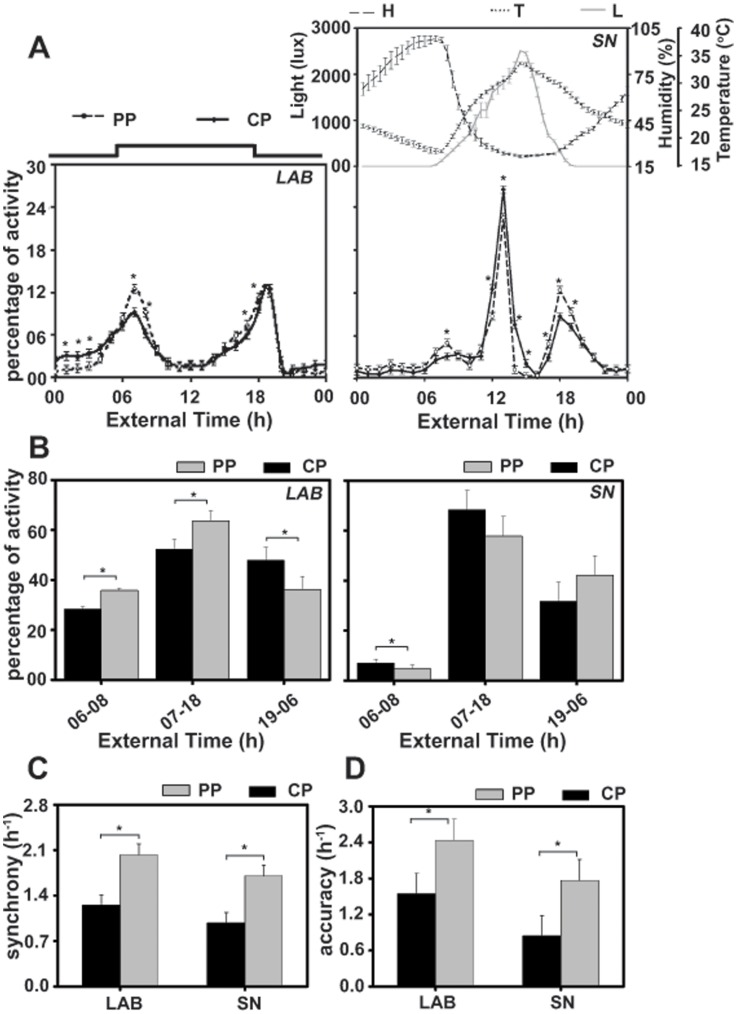
Flies from selected populations exhibit enhanced activity during the morning peak. (A) Activity profiles of flies from selected (*PP*) and control (*CP*) populations under laboratory (LAB) and semi-natural (SN) conditions. The percentage of activity averaged over 8–10 successive cycles is plotted along *y*-axis and time of the day in h along the *x*-axis. Flies from selected populations showed increased activity during the morning peak under LAB as well as in SN. The day-to-day variations in light, temperature and humidity are plotted as error bars (SEM) on their daily profiles. Other details are same as in Figure 2a. (B) Percentage activity during the morning peak, daytime, nighttime in flies from selected and control populations. Flies from *PP* were more active during the selection window under LAB and SN. (C, D) Synchrony and accuracy under LAB and SN. Enhanced synchrony and accuracy in the phase of morning activity peak in *PP* flies persisted under SN. About 32 flies were used for each of the four replicate populations under each environmental regime. The mean values of four replicates and variance in terms of 95% comparison interval (95%CI) were used to draw emergence profiles and error bars. All other details are same as in Figure 2.

Selection also altered the waveform of activity rhythm, consequently in LAB, morning activity peak and daytime activity of *PP* was enhanced and nighttime activity reduced compared to *CP* (Figure 4A, B). Under SN while morning and evening activity peaks of *PP* was significantly greater than *CP* (Figure 4A), daytime and nighttime activity of *PP* and *CP* did not differ (Figure 4B). Flies displayed a third peak of activity (afternoon peak; Figure 4A, B). The amplitude of afternoon peak in *PP* was significantly reduced compared to *CP* (Figure 4a). ANOVA revealed a statistically significant effect of T (*F_23,69_* = 90.04; *p*<0.0001), E×T (*F_23,69_* = 76.9; *p*<0.0001), P×T (*F_23,69_* = 3.64; *p*<0.0001), and E×P×T interaction (*F_23,69_* = 3.40; *p*<0.0001), whereas the effect of E (*F_1,3_* = 0.05; *p* = 0.8), P (*F_1,3_* = 0.09; *p* = 0.7) and E×P interaction was statistically not significant (*F_1,3_* = 0.09; *p* = 0.7). Post-hoc multiple comparisons using Tukey’s test revealed that, under LAB, activity during morning peak (ExT6-8) including the selection window (ExT7-8) and during evening peak (ExT16-18) was significantly enhanced and during nighttime (ExT0-4) significantly reduced in *PP* flies compared to *CP*. Similarly, under SN, activity of *PP* during morning peak (ExT7-8) and evening peak (ExT18-20) was significantly greater than *CP*. In addition, *PP* flies were significantly less active during afternoon (ExT13-16) compared to *CP* (Figure 4A). Persistence of increased activity during morning and evening peaks compared to controls under SN, suggests increased stability of activity rhythm in selected flies.

### Selected Populations Exhibit Enhanced Synchrony and Accuracy in Activity Rhythm Under SN

Under both conditions, *PP* flies showed enhanced synchrony and accuracy compared to *CP* (Figure 4C, D). ANOVA revealed a statistically significant effect of E (synchrony-*F_1,3_* = 17.1; *p*<0.02; accuracy-*F_1,3_* = 6.8; *p*<0.04), P (synchrony-*F_1,3_* = 10.1; *p*<0.04; accuracy-*F_1,3_* = 13.3; *p*<0.03), while the effect of E×P interaction was statistically not significant (synchrony-*F_1,3_* = 3.1; *p* = 0.1; accuracy-*F_1,3_* = 0.1; *p* = 0.9). Post-hoc multiple comparisons using Tukey’s test revealed that while synchrony and accuracy of activity rhythm in *PP* was significantly higher compared to *CP* under both LAB and SN (Figure 4C, D), it did not differ between SN and LAB. Persistence of enhanced synchrony and accuracy under SN indicates enhanced stability of activity rhythm in the selected flies.

### Stability of Emergence Rhythm Under Warm/Cold (WC) Cycle

**Figure 5:**
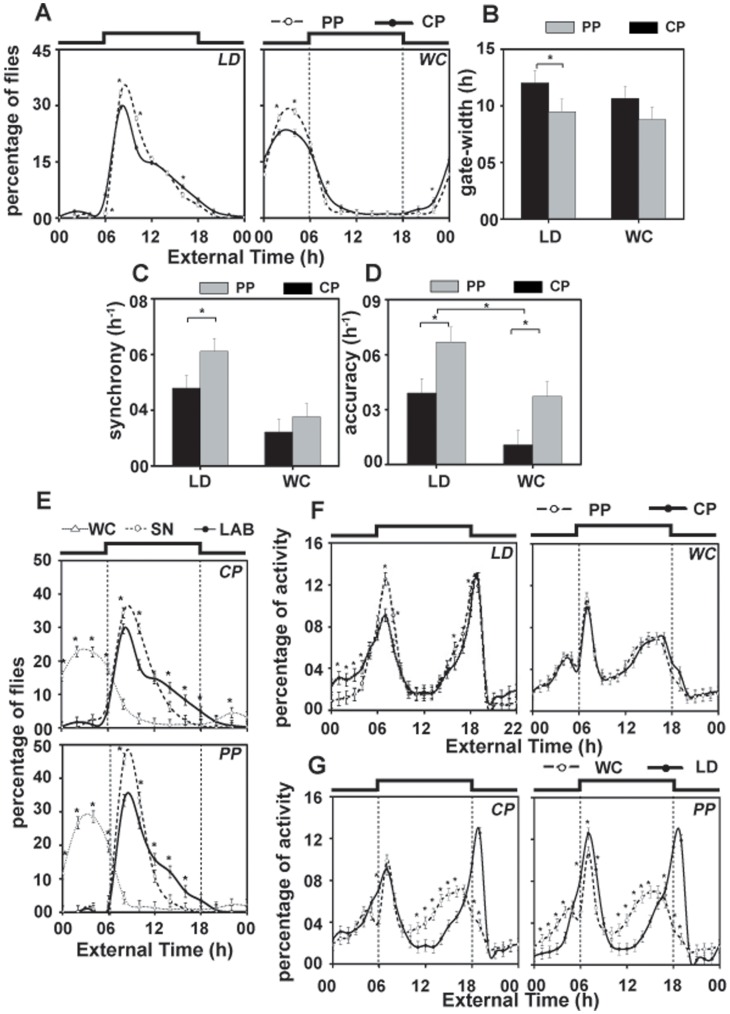
Circadian waveform of adult emergence and activity/rest rhythm under warm/cold (WC) cycles. (A) Waveform of emergence in the selected (*PP*) and control (*CP*) populations under12∶12 h light/dark (LD) and 29/25°C WC cycles. Although under WC, phase of emergence peak of both *PP* and *CP* was altered, *PP* flies continued to show enhanced emergence peak compared to controls. (B) Gate-width of emergence rhythm in *PP* and *CP* populations under LD and WC. Under LD, *PP* had narrower gate of emergence compared to controls whereas in WC the gate-width of *CP* and *PP* did not differ. (C, D) Synchrony, accuracy of emergence rhythm under LD and WC cycles. Although synchrony and accuracy is reduced under WC, the difference in accuracy between *PP* and *CP* is still maintained. (E) Comparison of emergence profile of *PP* and *CP* under SN, LD and WC. *PP* flies have greater amplitude of emergence peak under SN compared to LD and WC. (F, G) Activity rhythm of *PP*, *CP* flies under LD and WC. Under LD, *PP* flies exhibit enhanced morning activity peak compared to controls which did not persist in WC. In figure 5A, E, F and G the square-wave above the Figure represents 12∶12 h LD and WC cycles in LAB and vertical dotted lines represents the transition between warm and cool phase of WC, and in Figure 5E vertical dotted lines represent the transition between warm and cool phase of WC and dark and dark transition of LD. All other details are same as in Figure 2.

In order to study whether light or temperature cycles contributed more to the enhanced stability of emergence and activity rhythms under SN, we assayed these rhythms in the LAB under cycles of 12 h warm phase (29°C) and 12 h cool phase (25°C) in DD and compared it with those assayed under LAB LD at 25°C. While emergence peak occurred significantly earlier under WC compared to LD, *PP* flies displayed enhanced emergence peak compared to *CP* (Figure 5A). ANOVA on emergence data revealed a statistically significant effect of T (*F_11,33_* = 178.4; *p*<0.0001), E × T (*F_11,33_* = 807.5; *p*<0.0001), P × T (*F_11,33_* = 6.7; *p*<0.0001), and E×P×T interaction (*F_11,33_* = 9.1; *p*<0.0001), while the effect of E (*F_1,3_* = 1; *p* = 0.3) and P was statistically not significant (*F_1,3_* = 1; *p* = 0.3). Post-hoc multiple comparisons using Tukey’s test revealed that under WC, emergence in *PP* during ExT2-4 was significantly higher compared to *CP* (Figure 5A).

The tightness of emergence gate, evolved as a correlated response to selection, was not seen under WC (Figure 5B), which is associated with reduced synchrony in emergence rhythm in *PP*. Although accuracy in both populations was reduced in WC, *PP* flies had higher accuracy in emergence compared to *CP* (Figure 5C, D). ANOVA revealed a statistically significant effect of E (gate-width-*F_1,3_* = 16.7; *p*<0.02; synchrony*-F_1,3_* = 78.4; *p*<0.003; accuracy-*F_1,3_* = 22.4; *p*<0.02), P (gate-width-*F_1,3_* = 176.6; *p*<0.0009; synchrony*-F_1,3_* = 16.2; *p*<0.02; accuracy-*F_1,3_* = 21.6; *p*<0.01), and E×P interaction (accuracy-*F_1,3_* = 0.02; *p*<0.04), while the effect of E×P interaction on gate-width and synchrony was statistically not significant (gate-width-*F_1,3_* = 0.9; *p* = 0.4; synchrony-*F_1,3_* = 6.2; *p* = 0.08). Post-hoc multiple comparisons using Tukey’s test revealed that gate-width of *PP* did not differ from *CP* under WC, while in LD it was significantly narrower than *CP* (Figure 5B). Synchrony of emergence rhythm was reduced considerably in WC compared to LD, and *PP* flies had enhanced synchrony compared to *CP* in LD but not in WC (Figure 5C). Although, accuracy of emergence rhythm was significantly reduced under WC compared to LD, under both conditions it was significantly higher in *PP* compared to *CP* (Figure 5D). These results suggest that while within population features of emergence rhythm (amplitude and accuracy) of selected flies were higher under WC compared to controls, among population features (synchrony and gate-width) do not differ.

The amplitude of emergence rhythm was reduced under WC, and flies showed increased preference to emerge during the cold phase (Figure 5E). ANOVA on emergence data under SN, LD and WC showed a statistically significant effect of T (*F_11,33_* = 227.0; *p*<0.0001), E×T (*F_22,66_* = 252.0; *p*<0.0001), P×T (*F_11,33_* = 116.1; *p*<0.0001), and E×P×T interaction (*F_22,66_* = 106.3; *p*<0.0001), while the effect of E (*F_2,6_* = 0.8; *p* = 0.4) and P was statistically not significant (*F_1,3_* = 1.3; *p* = 0.3). Post-hoc multiple comparisons using Tukey’s test revealed that emergence peak of *PP* was significantly higher under SN compared to LD, while it was reduced under WC compared to LAB. The emergence rhythm seems to be differently modulated by external stimuli but *PP* flies continue to display enhanced robustness in emergence rhythm compared to *CP* under SN compared to laboratory LD and WC.

### Effect of Laboratory Warm/Cold (WC) Cycles on Activity/Rest Rhythm

*PP* flies displayed enhanced morning activity peak compared to *CP* under LD but not in WC (Figure 5F). Flies from both populations showed reduced amplitude of morning activity peak under WC compared to LD (Figure 5G). ANOVA on activity data revealed a statistically significant effect of T (*F_23,69_* = 199.8; *p*<0.0001), E×T (*F_23,69_* = 79.2; *p*<0.0001), P×T (*F_23,69_* = 7.2; *p*<0.0001), E×P×T interaction (*F_23,69_* = 9.7; *p*<0.0001), while the effect of E (*F_1,3_* = 0.05; *p* = 0.8) and P was not statistically significant (*F_1,3_* = 0.09; *p* = 0.7). Post-hoc multiple comparisons using Tukey’s test showed that while under LD, *PP* flies exhibited enhanced activity during morning (ExT6-8) and evening peaks (ExT16-18), activity of *PP* and *CP* flies did not differ under WC. Enhanced morning peak of activity in *PP* flies observed under LD did not persist under WC. This lack of any difference in the activity profiles of *PP* and *CP* under WC (Figure 5F) indicates that activity rhythm is more stable under LD cycles, whereas temperature cycles reduce the stability of activity rhythm in *PP* flies.

## Discussion

Accurate knowledge of time of the day is of paramount importance for organisms living under fluctuating environments. In the present study, we elucidated effect of semi-natural conditions on the stability (amplitude, synchrony and accuracy) of daily rhythms in *D. melanogaster* populations selected for emergence in a narrow window of time. In a previous study we had shown that such selection eliminated flies from both ends of the emergence waveform and favoured those that emerged during the selection window, and this eventually resulted in the evolution of increased stability in emergence and activity rhythms [21]. Such differences in stability between selected and control populations persisted under a wide variety of environmental conditions [22]. Interestingly, the enhanced peak and tighter waveform of emergence in selected flies as observed under LAB persists even more robustly in SN (Figures 1, 2), which suggests that natural conditions are more conducive for the expression of circadian phenotypes. In nature, emergence was mostly restricted to early hours of morning, which coincided with high humidity and low temperature (Figure 2B). Although emergence in the LAB and SN conditions was essentially at the same time, enhanced amplitude of the rhythm under SN suggests a role for other factors such as humidity and temperature in the regulation of amplitude and profile of emergence rhythm. Flies tend to emerge in association with gradually increasing light and/or temperature during dawn, suggesting that fly emergence in nature is regulated by multiple environmental cycles. Such emergence waveforms are consistent with those reported in a previous study [14], where emergence was found to be tightly gated around dawn, gradually tapering down towards the afternoon. However, this trend of consolidation of emergence under SN appears to not only additively contribute to the tightness and increase in the amplitude of daily rhythms, it seems to preferentially aid the selected populations more than the controls. Apparently, random fluctuations present in the SN environment are not nearly as strong a deterrent as perhaps high amplitude of light cycles or its waveform, to strengthen the selected phenotype even more than in native selection conditions.

The activity rhythm of flies entrained to natural zeitgebers with its morning peak synchronized to dawn and evening peak to dusk. Besides, flies displayed increased activity in the middle of the day, manifested in an additional afternoon peak [13]. The afternoon peak was significantly reduced in selected flies, however, they exhibited enhanced morning and evening activity peaks, and reduced variation in the phase of activity rhythm compared to controls (Figure 4). Therefore, flies selected for precise timing of emergence maintained greater stability in emergence and activity rhythms compared to controls even under SN, where it is challenged by stochasticity associated with diurnal changes. The afternoon peak is likely to be a stress response to bright light (∼2500 lux) on a warm day. This could be tested in future studies by giving flies access to natural light keeping temperature and humidity constant, or to conditions where the natural light alone is excluded, while flies experience natural variation in temperature, humidity and other factors.

Assessment of effects of laboratory light and temperature cycles on circadian entrainment, and on stability parameters of emergence and activity rhythms revealed that flies from selected populations has enhanced and accurate peak of emergence rhythm under LD and WC. However, under WC, synchrony and gate-width of selected flies did not differ from controls, which is probably because these flies were never exposed to temperature changes in the laboratory. This is reflected also in the lack of difference in robustness between selected and control flies when assayed under different ambient temperatures [22]. It is therefore likely that amplitude and stability in the phase of emergence peak are affected by light and temperature in a manner such that the differences between both populations persist, whereas light and temperature have differential effects on the gate-width and within-group synchrony. Whereas, for activity rhythm, light and temperature differentially affect selected populations such that robustness of morning activity peak of selected flies observed under LD, is diminished considerably under WC, rendering them as stable as controls. Since we find that differences in several features of rhythms between selected and control populations did not persist in WC, it is possible that enhanced stability of rhythms seen under SN is mostly owing to the effect of light. However, an important caveat to note here is that temperature in Bangalore during the course of the study varied between 18 to 30°C, but our choice of temperature for WC cycles was a square wave of 29∶25°C. We chose this temperature range considering the fact that the maximum and minimum temperatures under SN were prevalent only for short durations of time. We suspected that 12∶12 h square wave of high (30°C) and low (18°C) temperature may be stressful for flies. Therefore, although it is likely that stability of circadian rhythms in selected flies is primarily light-mediated under semi-natural conditions, a complex interaction of a multitude of environmental variables, perhaps acting in a hierarchical manner, regulate the stability of their circadian rhythms in nature. Besides light and temperature fluctuations, precision of circadian rhythms is also known to be affected by variations in photoperiod, which was reported to be higher around equinoxes, and lower during midsummer (in shrews) and during midwinter (in hamsters) [33]. Rigorous and systematic studies under natural conditions along with simulated laboratory conditions with varying light and temperature profiles mimicking natural conditions are required to elucidate how stability of circadian clocks is maintained in the face of daily and seasonally changing environments, and how interaction of these two environmental cycles influences stability of circadian entrainment in nature.

In summary, our study demonstrates the effect of semi-natural condition on stability of circadian rhythms in fruit fly *D. melanogaster* populations selected for emergence in a narrow window of time. Stability of circadian rhythms in flies from selected populations persisted even more robustly under semi-natural conditions displaying enhanced amplitude, synchrony and accuracy compared to controls, which suggests robustness of their time-keeping systems. Stability in emergence and activity rhythms of flies from the selected populations seems to stem from stability in their circadian entrainment primarily due to light, although role of temperature and other factors cannot be completely ruled out. To the best of our knowledge, this is the first study of its kind demonstrating the effect of day-to-day variations of several zeitgebers present in real natural environment on stability of circadian time-keeping.
